# Combined Linkage Mapping and BSA to Identify QTL and Candidate Genes for Plant Height and the Number of Nodes on the Main Stem in Soybean

**DOI:** 10.3390/ijms21010042

**Published:** 2019-12-19

**Authors:** Ruichao Li, Hongwei Jiang, Zhanguo Zhang, Yuanyuan Zhao, Jianguo Xie, Qiao Wang, Haiyang Zheng, Lilong Hou, Xin Xiong, Dawei Xin, Zhenbang Hu, Chunyan Liu, Xiaoxia Wu, Qingshan Chen

**Affiliations:** 1 College of Agriculture, Northeast Agricultural University, Harbin 150030, China; liruichao2018@126.com (R.L.); zhanguo7907@126.com (Z.Z.); zoyuanyuan@126.com (Y.Z.); xiejg519@163.com (J.X.); wqsoybean@163.com (Q.W.); zhenghaiyang_zhy@163.com (H.Z.); soybean_design@163.com (L.H.); xiongxin1905@163.com (X.X.); dwxin@neau.edu.cn (D.X.); hzb_net@126.com (Z.H.); cyliucn@126.com (C.L.); 2 Jilin Academy of Agricultural Sciences, Soybean Research Institute, Changchun 130033, China; j3994102@126.com

**Keywords:** PH and NNMS, BSA, QTL, haplotype analysis, soybean

## Abstract

Soybean is one of the most important food and oil crops in the world. Plant height (PH) and the number of nodes on the main stem (NNMS) are quantitative traits closely related to soybean yield. In this study, we used 208 chromosome segment substitution lines (CSSL) populations constructed using “SN14” and “ZYD00006” for quantitative trait locus (QTL) mapping of PH and NNMS. Combined with bulked segregant analysis (BSA) by extreme materials, 8 consistent QTLs were identified. According to the gene annotation of the QTL interval, a total of 335 genes were obtained. Five of which were associated with PH and NNMS, potentially representing candidate genes. RT-qPCR of these 5 candidate genes revealed two genes with differential relative expression levels in the stems of different materials. Haplotype analysis showed that different single nucleotide polymorphisms (SNPs) between the excellent haplotypes in *Glyma.04G251900* and *Glyma.16G156700* may be the cause of changes in these traits. These results provide the basis for research on candidate genes and marker-assisted selection (MAS) in soybean breeding.

## 1. Introduction

Soybean is an important food and oil crops because it is an important source of plant protein and edible oil [1,2]. Breeding both high quality and high-yield soybeans species remains an important goal pursued by breeders. Attaining high yield is not only determined by yield traits; plant types are also important aspects of yield potential [3]. Plant height (PH) and number of nodes on the main stem (NNMS) are two important quantitative traits affecting soybean plant type. In the 1950s, since the Green Revolution, great success has been achieved in reducing the height of plants to increase actual crop yield [4]. However, many studies have shown a significant positive correlation between PH and NNMS, reporting that decreasing PH reduces NNMS and weakens the increased yield to some extent [5,6]. Developing the application potential of two traits to cultivate the ideal soybean plant type cultivar with suitable plant height and a large number of nodes is a continued concern for breeders.

Mapping of quantitative trait loci (QTL) is an effective way to analyze quantitative crop traits. Molecular markers have been widely used in QTL mapping for various traits in soybean. Currently, approximately 230 and 37 QTLs have been identified for PH and NNMS in Soybase (www.soybase.org), respectively [5,7]. Previous studies have laid a solid foundation for studying genetic variation in soybean PH and NNMS, but most QTLs cannot be repeatedly verified in different populations or different environments. Therefore, the genetic mechanisms of soybean development still require an in-depth study.

Bulked segregant analysis (BSA) is a method of mixing individuals with extreme phenotypes in the isolated progeny of the population and comparing the polymorphisms between different extreme mixing pools and phenotypes for target gene localization [8]. Compared to traditional QTL mapping methods, BSA only requires consideration of a few extreme individuals in the population rather than the entire population, simplifying the sequencing process and significantly reducing the cost of sequencing and analysis [9]. In addition, the statistical power in QTL mapping is comparable to the entire population analysis, which is accomplished by using large populations, increased tail sizes, and high-density markers [10].

Previous studies have identified and cloned several genes that strongly influence PH and NNMS in plants, such as *Rht-D1b* [11] and *Rht24* [12] in wheat. In addition, SLR1 inhibits MOC1 degradation to coordinate PH in rice [13]. *OsmiR396d* is involved in the interaction network between Brassinolide (BR) and gibberellin (GA) signals, affecting PH [14]. Brachytic 2 is related to polar auxin transport in maize [15]. However, there are few reports on the genes controlling PH and NNMS in soybean. Expression of the Arabidopsis thaliana *BBX32* gene in soybean increases PH and NNMS [16]. Furthermore, *Cd1* and *Dt1* were identified to affect PH [17,18], and *GmBRI1* acts as a BR receptor, which affects the BR signal transduction pathway to reduce PH [19].

In this study, we used the chromosome segment substitution lines (CSSL) population constructed in our laboratory to locate QTLs of PH and NNMS by combining traditional QTL mapping and BSA traditional and validating QTLs in recombinant inbred lines (RIL) populations. We screened candidate genes in the QTL interval and validated the genes using haplotype analysis and real-time quantitative analysis.

## 2. Results

### 2.1. Phenotypic Data for PH and NNMS

**Figure 1:**
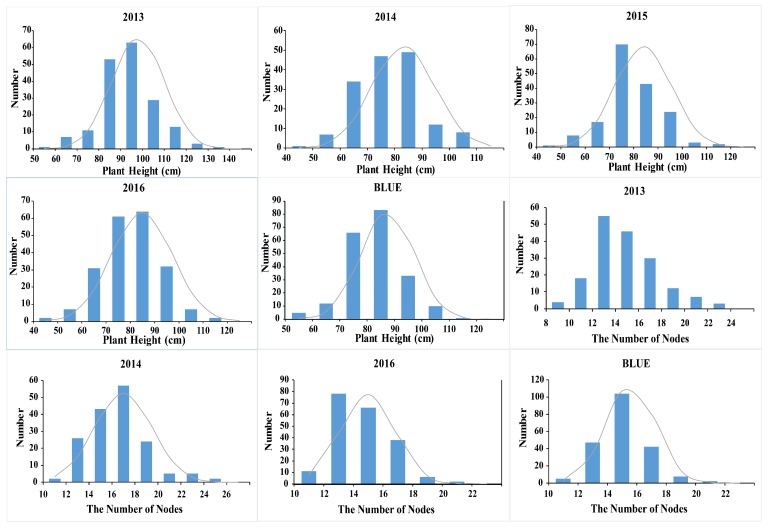
Frequency distribution of the soybean PH and NNMS in the CSSL population from 2013 to 2016.

The PH and NNMS phenotype of the CSSL population from 2013 to 2016 are shown in Table 1 and Figure 1. PH in 2013 was significantly higher than that in 2014–2016, exhibiting a minimum skewness of 0.03. In 2014, the phenotypic information of PH had a minimum standard deviation (SD) and a minimum kurtosis of 11.50 and 0.08, respectively. The broad-sense heritability of PH ranged from 0.69 to 0.77, and the higher broad-sense heritability indicated that most of the phenotypic variation was mainly controlled by genotypes. The differences in NNMS of the maternal parent were significant. The broad-sense heritability of NNMS was between 0.20 and 0.27, and the lower heritability indicated that NNMS was susceptible to environmental factors, with gene and environment together potentially affecting phenotypic variation. Correlation analysis results of the two trait phenotypes are shown in  Supplementary Table S1. In 2013–2016 (except for 2015), the Pearson correlation coefficient for PH and NNMS was between 0.364 and 0.398, indicating a weak correlation. The Pearson correlation coefficient between the BLUE is 0.495, which is moderately correlated. The phenotypic frequency distribution of the two traits exhibits an approximately normal distribution, and the heritability is highly reproducible, which is suitable for QTL mapping.

### 2.2. QTL Mapping Analysis for PH and NNMS in the CSSL Population

In total, 24 QTLs associated with the PH were detected from 2013 to 2016 and distributed over 13 chromosomes (Table 2). The PVE% (Phenotypic Variance Explained of the QTL) for all of the QTLs ranged from 3.05% to 21.70%, with the logarithm of odds (LOD) values between 3.08 and 15.89. qPH-k-1 had a maximum PVE of 21.70% with a LOD value of 9.66. qPH-j-1 had a maximum LOD of 15.89 while explained 17.04% of the variation. qPH-j-1, which had a minimum map distance of 0.02 Mb, was mapped between 32.76 and 32.78 Mb on Gm16, explaining approximately 17.04% of the variation and having an additive effect of −7.65. qPH-b1-1 had a maximum map distance of 1.03 Mb and was mapped between 24.41 and 25.44 Mb, explaining 3.89% of the variation with an additive effect of 9.80.

From 2013 to 2016 (except for 2015), 10 QTLs associated with NNMS were detected and distributed over 7 chromosomes (Table 3). The LOD values of all the QTLs above were from 2.68 to 12.19 and the PVE was between 4.61% and 20.12%. qMS-k-1 had a maximum PVE of 20.12% and a maximum LOD value of 12.19. qMS-j-1, which had a minimum map distance of 37.91 Kb, was mapped between 30.78 and 30.82 Mb on Gm16, thereby explaining approximately 8.18% of the variation and having an additive effect of −1.20.

### 2.3. Correlation Analysis by the Euclidean Distance (ED)

**Figure 2:**
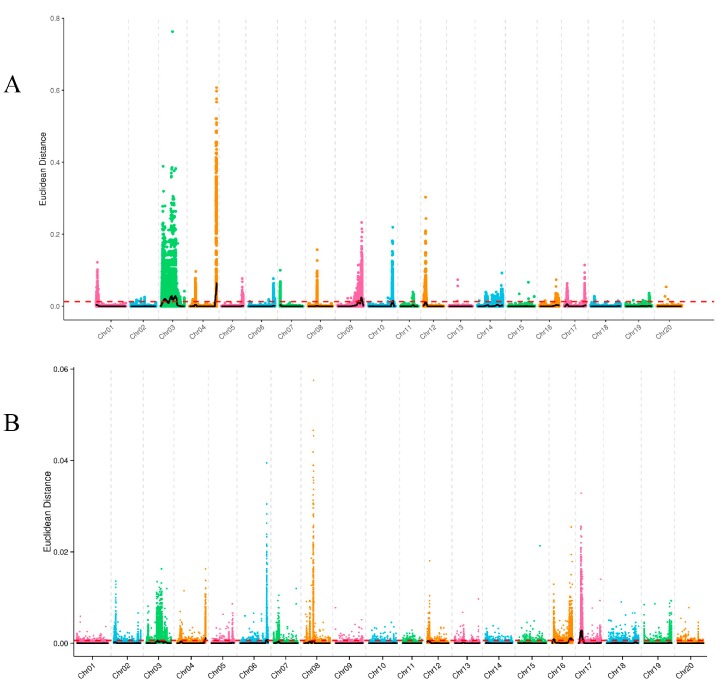
The distribution of Euclidean distance (ED)-associated values on chromosomes. Note: The abscissa is the chromosome name. The color points represent the ED value of each single nucleotide polymorphism (SNP) locus. The black line is the fitted ED value, and the red dotted line represents the significantly associated threshold. The higher the ED value, the better the correlation. (**A**) Analysis of ED-associated values in PH. (**B**) Analysis of ED-associated values in NNMS.

In this study, a total of 3,717,836 and 2,664,165 single nucleotide polymorphisms (SNPs) of PH and NNMS were used for correlation analysis by the Euclidean distance (ED) method. According to the rules of this method, we excluded SNP loci containing multiple alleles (those with more than one SNP within 5 bp) and SNP loci with identical genotypes between the two pools. Finally, we obtained 3,150,387 and 2,215,742 high-quality SNPs for PH and NNMS, respectively, and the support in both mixed pools was greater than 4 (Supplementary Table S2). After calculating the ED value, the correlation value was obtained using the local linear regression LOESS (locally weighted regression) method, and the candidate interval associated with the trait was determined by using 0.0129 and 0.0006 (median plus three standard deviations) as a standard threshold for PH and NNMS (Figure 2). Among them, there are six candidate intervals related to PH, which are distributed on four chromosomes: 3, 4, 9 and 10. There are eight related regions related to NNMS, which are distributed on four chromosomes: 4, 6, 16 and 17 (Table 4).

### 2.4. Consistency Interval Determination Combined with BSA and ICIM

**Figure 3:**
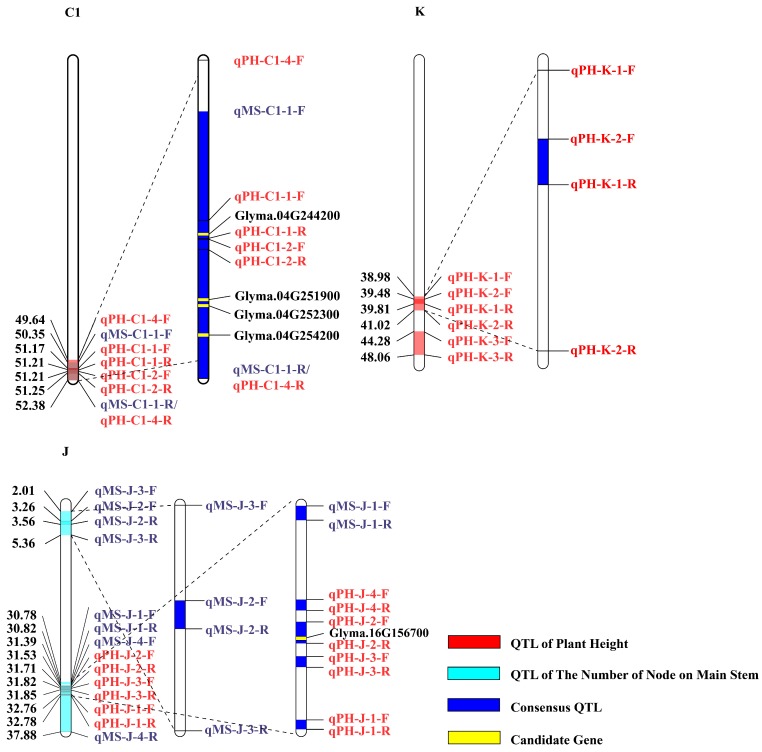
Distribution of consistent QTLs and candidate genes. Note: red represents the QTL of PH; light blue represents the QTL of NNMS; navy blue represents the consensus QTL; yellow represents the candidate gene.

For these QTLs, we obtained several relatively stable QTLs and narrowed the confidence interval of QTLs by comparing the overlapping intervals of QTLs from the BSA and ICIM (Inclusive Composite Interval Mapping) methods. In terms of PH, two stable QTLs were identified by comparing the overlapping intervals of both methods, and they were located on chromosome 4 from 51.16 Mb to 51.25 Mb and on chromosome 9 from 39.48 Mb to 39.81 Mb. In terms of NNMS, a significant QTL interval was also obtained after comparison, it was located on chromosome 16 from 3.26 Mb to 3.56 Mb. Interestingly, when we integrated the QTL of PH and NNMS for a comprehensive analysis, there were multiple significant QTLs identified that may affect both two traits at the same position. A region from 50.35 Mb to 52.38 Mb on chromosome 4 was detected multiple times by these two methods. From 30.78 Mb to 32.78 Mb on chromosome 16, it is an important region where the QTLs were repeatedly positioned five times for the two traits. Ultimately, we obtained a total of 8 carefully selected QTLs for subsequent analysis (Figure 3).

### 2.5. Mining Candidate Genes

A total of 335 candidate genes were screened in 8 major QTL intervals (Supplementary Table S3), 5 of which were related to plant growth and development were found based on the annotation information (Supplementary Table S4).

*Glyma.04G252300* is in the KEGG pathway (pathway ID K13946) associated with the auxin influx carrier, which was involved in the transport of auxin. The homologous gene in Arabidopsis is *AT1G77690,* which encodes an auxin influx carrier LAX3, (Like Aux1), that promotes lateral root emergence [20]. *Glyma.04G254200* belongs to the auxin response factor family and affects plant growth by activating or inhibiting transcription to regulate expression of auxin genes. Its homologous gene in Arabidopsis, *AT1G77850*, is associated with the formation of auxin response factor 17, the transcription factor that regulates auxin-responsive gene expression [21]. *Glyma.04G244200* occurs in the KEGG pathway (pathway ID K05282) and is associated with the synthesis of gibberellin 20, which encodes gibberellin 20-oxidase. The *SD1* gene in the rice caused mutations in the encoded GA20ox-2, reducing the amount of active GA in the leaves and resulting in a short plant [22]. *Glyma.16G156700* and *Glyma.04G251900* belong to the GRAS family (gibberellic-acid insensitive (*GAI*), repressor of GAI (*RGA*), and scarecrow (*SCR*)), which are major players in gibberellin (GA) signaling and regulate various aspects of plant growth and development. *AT3G54220* and *AT4G08250* are their homologous genes in Arabidopsis thaliana, respectively. *AT4G08250* encodes nodulation-signaling pathway 2 protein-like (NSP2), which is closely related to nodulation of legumes [23].

### 2.6. Expression Analysis of Candidate Genes

**Figure 4:**
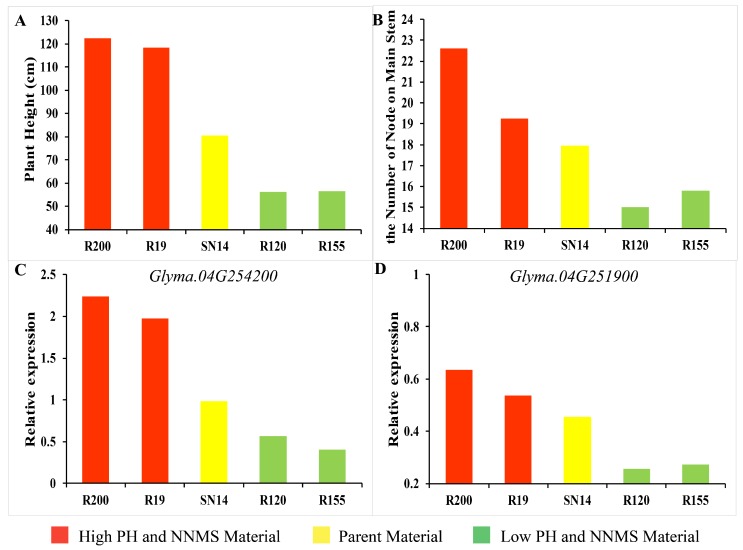
Expression of candidate gene by RT-qPCR. (**A**) Phenotypes of PH for R15, R200, SN14, R120, and R155. (**B**) Phenotypes of NNMS for R15, R200, SN14, R120, and R155. (**C**) Expression of *Glyma.04G254200*. (**D**) Expression of *Glyma.04G251900*.

Three of the candidate genes (*Glyma.04G244200*, *Glyma.16G156700*, and *Glyma.04G252300*) were barely expressed in soybean stems. This result was consistent with the relative expression levels of genes found in Phytozome (https://phytozome.jgi.doe.gov/pz/portal.html). The four-year average PH and NNMS of these five materials are shown in Figure 4A,B. R19 and R200 had outstanding phenotypic values compared with the other three materials, while in Figure 4C,D, *Glyma.04G254200* and *Glyma.04G251900* also had higher expression levels in these lines. In contrast, R120 and R155 had lower phenotypic values, and the relative expression of candidate genes was also significantly lower than in other materials. The higher expression levels of the genes in the stems matched the higher PH and NNMS of the soybean material. The reverse was also true, revealing that these genes may play a positive role in soybean PH and NNMS at this stage. *Glyma.04G244200* had the highest relative expression compared to other genes, suggesting that it may play a more significant role at this stage. These results indicate that the expression of candidate genes is closely related to the development of soybean PH and NNMS.

### 2.7. Haplotype Analysis

**Figure 5:**
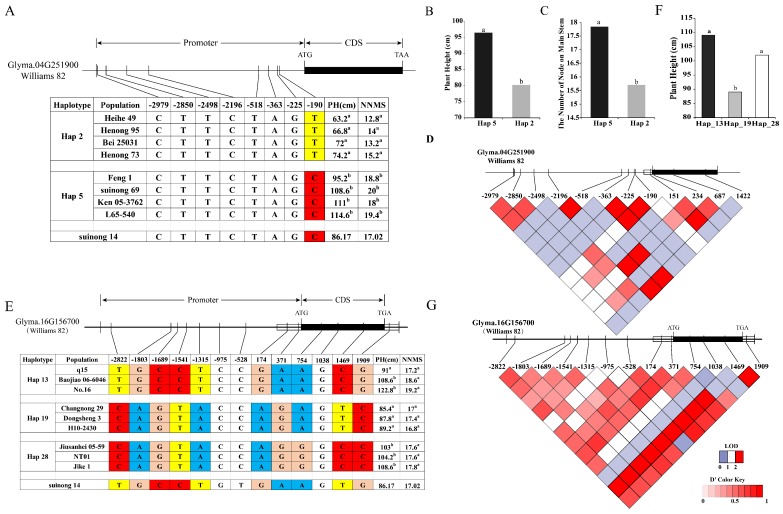
Haplotype analysis of the candidate gene. (**A**) Haplotype analysis of *Glyma.04G251900* from 92 soybean resource. (**B**) PH of Hap-2 and Hap-5. (**C**) NNMS of Hap-2 and Hap-5. (**D**) linkage disequilibrium (LD) analysis of SNPs located on *Glyma.04G251900*. Red from light to dark represents the degree of linkage between SNPs. (**E**) Haplotype analysis of *Glyma.16G156700* from 92 soybean resource. (**F**) PH of Hap-13, Hap-19, and Hap-28. (**G**) LD analysis of SNPs located on *Glyma.16G156700*. ^a^, ^b^: Different letters represent significant differences between each other at the 0.05 level.

Haplotype analysis of candidate genes in ninety-two germplasm resources using Dnasp5.0 software was performed next. Phenotypic data from the germplasm resources followed the test results in 2018 (Supplementary Tables S5 and S6). The PH of germplasm resources ranged from 62.4 cm to 140.4 cm, and the range of NNMS was from 12.6 to 24.8. There were abundant variations in both PH and NNMS in the natural resource, meeting the candidate haplotype analysis requirements. The results showed that *Glyma.04G244200*, *Glyma.04G251900*, and *Glyma.16G156700* had two or more excellent haplotypes (>5% of the population) (Supplementary Table S7), while *Glyma.04G252300* and *Glyma.04G254200* had only one excellent haplotype in the germplasm resource. Based on phenotypic data of ninety-two germplasm resource populations in PH and NNMS, the ANOVA method was used to analyze the differences between the phenotypes of the excellent haplotypes. Significant differences in PH and NNMS were showed only between the excellent haplotypes of *Glyma.04G251900* and *Glyma.16G156700* (Supplementary Table S8). *Glyma.04G251900* had two excellent haplotypes, Hap-2 and Hap-5, which exhibited significant differences in PH and NNMS (Figure 5B,C). Hap-2 contained 9 resources with a reduced average of PH and NNMS, and Hap-5 included 55 resources and the average PH and NNMS were significantly higher than those in Hap-2. SNP-190 was the only distinct SNP between Hap-2 and Hap-5 (Figure 5A), and SNP-190 carries a cis element (ARR1) [24] that changes according to PLACE analysis. According to linkage disequilibrium (LD) analysis, there is linkage between SNP-190 and SNP-225 (Figure 5D), and SNP-225 encodes the cis element ARR1 as well, potentially representing the cause for differential in gene function. *Glyma.16G156700* has four excellent haplotypes with significant differences between Hap-19 with Hap-19 and Hap-13 and between Hap-19 and Hap-28 with respect to PH according to ANOVA based on phenotypic values and haplotypes (Figure 5F). Analysis of SNPs in the promoter and CDS (Coding sequence) regions revealed that there were two SNP differences between the Hap-19 and Hap-28 in the CDS region (Figure 5E). The change in SNP1469 was a nonsense mutation, and SNP754 resulted in amino acid changes (W > L), potentially leading to changes in the structure and function of the gene. There are multiple SNP differences between Hap-19 and Hap-13 (Figure 5E), of which SNP-1803 and SNP-1689 lead to cis element ethylene responsive element [25] and CAAT-box [26] changes according to PLACE and Plant CARE analysis, and linkage disequilibrium analysis shows linkage imbalance between SNP-1803 and SNP-1689 (Figure 5G). This may be the cause of differences in traits but requires more in-depth research to confirm.

## 3. Discussion

CSSL is a high-generation backcross population constructed by using the parental hybrid F_1_ generation and the recurrent parent to perform multiple backcrossing and combining molecular marker-assisted selection of the donor parent chromosome fragment. The genetic background of CSSL is usually derived from the recurrent parent and contains only one or a few introduced fragments of the donor parent, which greatly reduces the interference from the genetic background. It is an ideal material for QTL positioning due to improved QTL accuracy. At present, many useful results have been achieved in rice [27,28], maize [29], cucumber [30], and *Gossypium hirsutum* [31]. There are also extensive studies on 100-seed weight [32,33], seed shape [34], and drug resistance [35] in soybean. During the long process of crop domestication, each generation has only the best seeds to form the next generation, which reduces the genetic diversity of the entire genome in the long run [36]. In this study, the wild soybean variety ZYD00006 was used as the donor parent to construct the CSSL population, and genes were introduced to broaden the genetic resources within the population. Each strain in the population contains only one or a few introduced fragments, facilitating rapid and more accurate localization of the main QTL due to the small genetic difference.

PH and NNMS play major roles in controlling plant type and affecting crop yield. Previous studies have shown a positive correlation between PH and NNMS [6]. Thus, the same QTL may affect both traits due to pleiotropic effects. In the present study, we identified multiple QTLs that affect both PH and NNMS, for example, qPH-C1-3 and qMS-C1-1 have coincident positions at 50.35 Mb–52.38 Mb on Gm04. Among them, the area between 51.16 Mb and 51.24 Mb was identified multiple times and maybe an important site related to growth and development. Lee et al. also located a PH QTL near this location [37]. However, most QTLs only work on PH or NNMS, similar to qPH-d1a-1, which only affects PH and qMS-d1a-1, which is only related to NNMS. These results suggest that the genetic mechanisms of PH and NNMS may be similar but not identical.

Currently, the number of QTLs for PH and NNMS that can be queried in the Soybase database (http://www.soybase.org) is 230 and 39, respectively. Yin et al. interrogated 159 QTLs for PH on the Soybase database and 23 QTLs determined by the 8-year phenotypic data of the RIL population for meta-analysis, obtaining 36 Meta QTLs [38]. In the present study, we identified a total of 24 and 10 QTLs for PH and NNMS, including 10 and 6 QTLs of that completely coincided or intersected with previous studies and 4 QTLs for PH that matched Meta QTL [39,40,41,42]. In addition, the resulting QTLs had smaller confidence intervals, illustrating the reliability and accuracy of these results. Among these, 14 and 4 QTLs did not appear in previous results for PH and NNMS, indicating that they are newly identified QTLs and providing an important basis for understanding plant genetic structure.

Currently, there have been multiple reports concerning the control of PH, *Dwarf5* (*d5*) has defects in terpene synthase (TPS) enzymes involved in the early step of GA biosynthesis that result in reduced PH, and *Dwarf18* causes a mutation in the *GA3ox* gene, resulting in the dwarfism [43,44]. Both of these mutations reduce the GA content in plants by affecting the GA biosynthetic pathway or signal transduction that dwarfs the plants. *Glyma.04G244200*, *Glyma.16G156700* and *Glyma.04G251900* are involved in the GAs pathway. *Glyma.04G244200* encodes the key enzyme GA20ox in the GA synthesis pathway. *GA20ox*, *GA3ox*, *GA2ox* and *EUI1* together form a feedback transcriptional control mechanism to maintain the homeostasis of GA content in plants. Inhibition or loss of function of the *GA20ox* gene results in dwarfing of the plant [45,46]. This may be an important candidate gene for PH in soybean. Both *Glyma.16G156700* and *Glyma.04G251900* contain a common GRAS domain. Zhou et al. isolated a novel GRAS transcription factor, *SlGRAS26*, in *Solanum lycopersicum*, illustrating that its downregulation generated pleiotropic phenotypes, including reduced PH [47]. The homologous gene of *Glyma.16G156700* is *AT3G54220*, which encodes the SCARECROW-like protein, and Zhang et al. found that *SCL3* can promote gibberellin signaling by antagonizing the master growth repressor DELLA in Arabidopsis [48]. These results suggest that these three candidate genes may affect PH and NNMS by regulating the synthesis of gibberellin. In recent years, results have shown that *PIN* [49,50], *Actin7* [51], and *BZR1* [52] control the synthesis and transport of auxin and brassinolide to affect PH and NNMS. *Glyma.04G252300* and *Glyma.04G254200* encode the auxin influx carrier LAX3, and auxin response factors are involved in the regulation of auxin expression. AUX1 and LAX3 participate in the interaction of auxin and ethylene in Arabidopsis, affecting the expression of auxin [53]. ARFs (Auxin Response Factor) are a family of functionally distinct response factors that regulate auxin to complete various plant growth and development processes [54]. MiR160 is a negative regulator of *ARF17*, which regulates the expression of auxin and affects the production of adventitious roots [55,56]. These results suggest that PH and NNMS may be affected by complex plant hormone regulatory networks, laying the foundation for our study of the molecular mechanisms of PH and NNMS.

Herein, we identified candidate genes that may affect PH and NNMS and discussed gene functions that are involved in the synthetic transcription pathway of plant hormones and play an important role in PH and NNMS. Further work is required to elucidate the molecular mechanisms of these candidate genes.

## 4. Materials and Methods

### 4.1. Plant Material Planting and Phenotypic Determination

A total of 194 whole-genome chromosome segment substitution lines (BC_3_F_n_, BC_4_F_n_) were constructed by crossing “suinong14” (the main cultivars of Heilongjiang, China) and “ZYD00006” (wild soybean resources) and subsequent backcrossing with suinong14 (Supplementary Figure S1) [33]. Based on this study, the number of CSSL populations was increased to 208 by using SSR markers to identify uncovered fragments. During 2012–2016, the CSSL populations were planted in Xiangyang Farm, Harbin, Heilongjiang Province (Harbin, latitude 45°450” N, longitude 126°380” E). A randomized complete block design with three replicates was used over 4 years. The lines were grown in a one-row plot with 60-cm row spacing and a 6-cm space between plants. The rows were 5 m long, with approximately 80 plants per row. In October of each year, five individuals with the same growth status were selected in each line for PH and NNMS measurements in the field, and field management followed the common agricultural practice. PH (cm) is the average length from the cotyledonary node to the top of the mature plant [57]. NNMS indicates the number of nodes from the cotyledonary node to the top of the main stem. The linear mixed model is used to integrate the phenotypic data from different years, the individual’s genotype effect is used as the fixed effect and the year change is used as the random effect. The lme4 in R software is used to calculate the best linear unbiased estimator (BLUE) of each individual. The mixed linear model is calculated according to the following formula:

(1) y=Xβ+Yγ+ε

where “*X*” represents the fixed effect; “*β*” represents fixed-effects parameter estimates; “Z” represents the random effect; “*γ*” represents random-effects parameter estimates; “*ε*” represents the errors. Based on the analysis of variance, the AOV model in QTL ICIMapping Version 4.1 (Beijing, China) was used to determine the broad-sense heritability [58,59].

(2) $$h^{2}=r\sigma_{g}^{2}/\left(\sigma_{e}^{2}+r\sigma_{g}^{2}\right)$$

where “*r*” represents the number of repetitions; “$\sigma_{g}^{2}$”represents the genetic variance; “$\sigma_{e}^{2}$”represents the environmental variance.

### 4.2. QTL Analysis in the CSSL Population

ICIMapping 4.1 was used for QTL positioning by using the CSSL module. The presence of QTL based on a LOD value greater than 2.5 was determined. QTL naming was based on the method of McCouch [60]. The QTL name is constructed as follows: q+ trait + LG or LG number + QTL number.

### 4.3. DNA Extraction and Pool Construction

Combining the phenotypic data of the four years, 30 (>10% of the total population [8,10]) extreme phenotype individuals were selected from the CSSL population according to the four extreme performances (high PH, low PH, high NNMS and low NNMS) (Supplementary Table S9). Fresh leaves were cryopreserved using liquid nitrogen, DNA of leaves was extracted using the CTAB (cetyltrimethylammonium Ammonium Bromide) plant tissue DNA extraction method, DNA concentration and purity were measured using a Nano Drop 2000C (Sunnyvale, California, USA) ultra-micro spectrophotometer and 1.5% agarose gel electrophoresis. The DNA was randomly disrupted by ultrasonic disruption, and the DNA fragment was end-repaired, the 3′ end was filled with a base, and the sequencing linker was added. The DNA fragment was enriched by PCR amplification, and the product was purified to remove the contamination of the linker. The construction of the sequencing pool library was completed. The sequencing library constructed above was used for pair-end sequencing (each end 150 bp) on an Illumina HiSeq 2500 platform (San Diego, CA, USA) using the normal protocol at Beijing Biomarker Technologies Corporation (http://www.biomarker.com.cn).

### 4.4. Data Analysis and Filtering

To ensure the quality of information analysis, the following methods were adopted: the sequence containing the adapter was removed; the paired-end reads of N > 10% on the sequence were removed (the specific base type cannot be determined); the low-quality reads were removed (the number of bases with a mass value of Q ≤ 10 is more than 50% of the entire read); and the raw data was filtered. The genome of the soybean variety Williams 82 was used as a reference genome, and the short sequence obtained from the second-generation high-throughput sequencing was compared with the reference genome using BWA software (https://sourceforge.net/projects/bio-bwa/) [61]. According to the positioning results of Clean Reads on the reference genome, the Picard tool was used (http://sourceforge.net/projects/picard/) to remove the duplicates. After the local realignment and correction of base quality values were performed by GATK software (Cambridge, Massachusetts, USA) [62], SNPs were detected to ensure the accuracy of the detected SNP. Finally, SNP clusters were filtered out (filtered out if there are 2 SNPs within 5 bp); SNPs near indels were filtered out (SNPs filtered within 5 bp near an indel); Filtering adjacent indel was used (two Indel distances less than 10 bp filtered) [63]; and the SNPs were rigorously filtered to obtain the final SNP locus set.

### 4.5. Correlation Analysis by the Euclidean Distance (ED)

The Euclidean Distance (ED) algorithm is a method for identifying significant differences between mixed pools using sequencing data and evaluating areas associated with traits [64]. In theory, there are differences in the target trait related sites between the two pools constructed by the BSA, while other sites tend to be consistent, and the ED value should tend to zero. The formula for the ED method is as follows, and the larger the ED value, the larger the difference between the two mixed pools.

(3) $$ED=\sqrt{{\left(A_{mut}-A_{wt}\right)}^{2}+{\left(C_{mut}-C_{wt}\right)}^{2}+{\left(G_{mut}-G_{wt}\right)}^{2}+{\left(T_{mut}-T_{wt}\right)}^{2}}$$

*A_mut_* is the frequency of the *A* base in the mutation pool, and *A_wt_* is the frequency of the *A* base in the wild pool; *C_mut_* is the frequency of the *C* base in the mutation pool and *C_wt_* is the frequency of the *C* base in the wild pool; *G_mut_* is the frequency of the *G* base in the mutation pool and *G_wt_* is the frequency of the *G* base in the wild pool; *T_mut_* is the frequency of the *T* base in the mutation pool and *T_wt_* is the frequency of the *T* base in the wild pool. In the analysis, SNP sites with different genotypes between the two pools were used to calculate the depth of each base in different pools, and the *ED* value of each locus was calculated. This study used the median plus three standard deviations as the correlation value to eliminate background interference. A region above the threshold is selected as the region related to the trait according to the threshold (median plus three standard deviation).

### 4.6. Gene prediction of Major QTL Intervals

The soybean gene in the target segment was identified using the “Williams 82.a2.v1” genome as the reference genome. All genes in the QTL interval were extracted, and gene functions were annotated by the Kyoto Encyclopedia of Genes and Genomes (KEGG) (https://www.kegg.jp/), gene ontology (GO) (https://www.ebi.ac.uk/QuickGO/), and Pfam (https://pfam.xfam.org/) databases. Finally, the candidate genes were obtained.

### 4.7. RT-qPCR Analysis of Candidate Genes

Based on the phenotypes of PH and NNMS, we selected extremely high materials R19 (the PH is 118.25 cm, the NNMS is 19.25) and R200 (122.4 cm, 22.6) and extremely small materials R120 (56.2 cm, 15) and R155 (56.4 cm, 15.8) and SN14 (81.0 cm, 18.1). There are significant phenotypic differences at the *P* = 0.05 level between extremely high line and extremely small lines on PH and NNMS (Supplementary Tables S10 and S11). Extraction of shoot tip tissue from the ternate compound-leaf stage for RNA extraction using TRIzol Reagent (Invitrogen, 15596-026, Carlsbad, CA, USA) was performed. Reverse transcription of extracted RNA into cDNA using the Tianhe Real-time quantitative PCR (RT-qPCR) kit was performed using SYBR qPCR Mix (Vazyme, Q711, Vazyme biotech, Nanjing, China) on the Light Cycler 480 System (Roche, Roche Diagnostics, Basel, Switzerland). The expression levels of candidate genes were calculated with *GmUKN1* as an internal reference according to the following formula [65]:

(4) *Relative Expression* = 2^ΔCt^, [ΔCt = Ct (*GmUKNI*) − Ct (target genes)]

The qRT-PCR primer sequences specific for candidate genes were designed using Primer Premier 5.0 (http://www.premierbiosoft.com/primerdesign/index.html).

### 4.8. Haplotype Analysis

Ninety-two germplasm resources from all over China were used for haplotype analysis due to their rich genetic variation and large fluctuation range of PH and NNMS. Ninety-two germplasm resources were also planted in Xiangyang Farm, Harbin. Planting methods and field management were the same as for the CSSL population. The genomic sequence of the candidate gene was proposed on the Phytozome12 website (https://phytozome.jgi.doe.gov/pz/portal.html), including the 5′UTR, the upstream 3000 bp promoter region sequence, CDS sequence, intron sequence, and 3′UTR sequence information. The sequence information of the candidate gene and the 92 soybean germplasm resources resequencing genomic sequence information were subjected to local BLAST analysis to obtain SNP information of the candidate gene in the germplasm resource population. In this study, Dnasp5.0 software was used to analyze the haplotype distribution of candidate gene SNP sequences in germplasm resource populations, and the excellent haplotypes (the number of cultivars belonging to the haplotype exceeded 5% of the total) were screened out (https://haploview.software.informer.com/4.2/). Using the Haps Format module in Haploview 4.2 software (Cambridge, Massachusetts, USA) to analyze the excellent haplotype sequence information of candidate genes, ANOVA analysis of candidate gene haplotypes and phenotypes using SPSS 17.0 software (Armonk, New York, USA) was used to determine the effect of each haplotype on phenotype. PLACE (Plant cis-acting regulatory DNA elements (https://www.dna.affrc.go.jp/PLACE/)) and Plant CARE (Plant promoters and cis-acting regulatory elements (http://bioinformatics.psb.ugent.be/webtools/plantcare/html/)) softwares were used to query the function of plant promoter elements.

## 5. Conclusions

We combined linkage mapping and BSA to identify QTL and detected 8 consistent intervals. According to the gene annotation of the QTL interval, five of which were associated with PH and NNMS. RT-qPCR and haplotype analysis showed that they may be candidate genes that affect PH and NNMS. These results provide the basis for research on candidate genes and marker-assisted selection (MAS) in soybean breeding.

## Figures and Tables

**Table 1:** Phenotypic statistical of chromosome segment substitution lines (CSSL) populations in plant height (PH) and number of nodes on the main stem (NNMS) from 2013 to 2016.

Traits	Year	Parent	Population						
			Max ^a^	Min ^b^	Mean ^c^	SD ^d^	Kurtosis ^e^	Skewness ^f^	H^2 h^
PH	2013	101.75	135.83	58.50	92.92	12.16	0.96	0.03	0.76
	2014	77.17	108.80	47.50	78.13	11.50	0.08	0.07	0.69
	2015	87.93	116.20	46.25	78.92	11.64	0.85	0.16	0.72
	2016	77.83	118.75	28.00	79.77	12.60	2.15	−0.55	0.77
	BLUE	86.34	113.02	54.78	82.48	9.62	0.98	0.41	-
NNMS	2013	20.35	24	8	14.31	2.78	0.60	0.71	0.27
	2014	16.30	25	11	15.97	2.54	1.04	0.77	0.26
	2016	14.40	20	10	13.96	1.85	−0.08	0.54	0.20
	BLUE	16.58	25	11	15.65	2.04	1.04	2.59	-

^a^ Max (cm): The maximum of the phenotypic data from the CSSLs; ^b^ Min (cm): The minimum of the phenotypic data from the CSSLs; ^c^ Mean (cm): The average values of the phenotypic data from the CSSLs; ^d^ SD: Standard deviation of the phenotypic trait; ^e^ Kurtosis: Kurtosis of the phenotypic trait; ^f^ Skewness: Skewness of the phenotypic trait; ^h^ H^2^: The broad-sense heritability of the phenotypic trait.

**Table 2:** The result of quantitative trait locus (QTL) in PH.

Year	QTL	Chromosome ID	Additive	LOD	PVE (%)	Start Position (bp)	End Position (bp)	Size (Mb)
2013	qPH-m-1	Gm07	−7.74	4.61	4.33	3,900,022	3,934,584	0.03
2013	qPH-o-1	Gm10	−8.45	6.61	6.36	2,643,396	2,728,613	0.09
2013	qPH-o-2	Gm10	9.85	10.94	11.06	45,348,039	45,606,087	0.26
2013	qPH-j-1	Gm16	−7.65	15.89	17.04	32,755,660	32,778,805	0.02
2013	qPH-g-1	Gm18	6.22	5.91	5.63	57,614,442	57,682,794	0.07
2013	qPH-i-1	Gm20	4.61	3.34	3.09	33,541,216	33,591,497	0.05
2014	qPH-k-1	Gm09	11.42	9.66	21.70	38,984,325	39,808,126	0.82
2014	qPH-o-3	Gm10	−6.76	3.31	6.80	3,044,123	3,089,804	0.05
2014	qPH-d2-1	Gm17	6.68	3.24	6.65	38,033,581	38,140,597	0.11
2015	qPH-c1-1	Gm04	6.01	5.16	10.22	51,165,545	51,205,951	0.04
2015	qPH-j-2	Gm16	−6.63	4.26	9.14	31,527,228	31,708,328	0.18
2016	qPH-d1a-1	Gm01	−5.68	6.47	6.88	4,956,352	5,012,034	0.06
2016	qPH-d1a-2	Gm01	4.14	4.06	4.26	27,204,683	27,663,584	0.46
2016	qPH-c1-2	Gm04	7.05	12.74	13.97	51,206,176	51,248,063	0.04
2016	qPH-m-2	Gm07	−6.92	6.90	7.36	36,657,129	36,690,007	0.03
2016	qPH-o-4	Gm10	−5.82	3.08	3.13	2,733,103	2,932,573	0.20
2016	qPH-b1-1	Gm11	9.81	3.80	3.89	24,409,526	25,442,397	1.03
2016	qPH-e-1	Gm15	−5.14	4.50	4.72	14,578,705	14,885,298	0.31
2016	qPH-j-3	Gm16	−5.34	6.51	6.98	31,822,329	31,845,684	0.02
2016	qPH-l-1	Gm19	−5.10	6.36	6.78	46,391,451	46,417,246	0.03
BLUE	qPH-c2-1	Gm06	−7.96	11.71	9.74	3,612,257	3,655,776	0.04
BLUE	qPH-m-3	Gm07	−5.62	5.51	4.26	4,023,721	4,389,344	0.37
BLUE	qPH-j-4	Gm16	−3.34	6.26	4.89	31,484,839	31,506,867	0.03
BLUE	qPH-g-2	Gm18	6.27	7.63	6.05	54,901,888	54,960,317	0.06

**Table 3:** The result of QTL in NNMS.

Year	QTL	Chromosome ID	Additive	LOD	PVE (%)	Start Position (bp)	End Position (bp)	Size (Mb)
2014	qMS-d1a-1	Gm01	2.09	2.87	6.97	50,129,391	50,289,706	0.16
2014	qMS-a2-1	Gm08	−1.25	2.68	6.50	22,007,790	22,273,422	0.27
2014	qMS-j-1	Gm16	−1.20	3.22	8.18	30,783,034	30,820,945	0.04
2016	qMS-c2-1	Gm06	1.92	4.40	5.90	4,184,901	4,275,666	0.09
2016	qMS-c2-2	Gm06	−3.61	10.37	15.07	10,205,331	10,819,289	0.61
2016	qMS-h-1	Gm12	1.62	6.32	8.91	1,733,331	1,854,712	0.12
2016	qMS-j-2	Gm16	−1.34	3.85	5.19	3,261,870	3,556,117	0.29
2016	qMS-l-1	Gm19	1.76	3.72	5.00	3,626,636	3,814,283	0.19
BLUE	qMS-k-1	Gm09	2.00	12.19	20.12	39,484,325	39,808,126	0.82
BLUE	qMS-h-2	Gm12	0.95	5.42	8.47	2,635,139	2,693,348	0.06

**Table 4:** QTL mapping of soybean PH and NNMS by bulked segregant analysis (BSA).

Trait	QTL	Chromosome ID	Start Position (bp)	End Position (bp)	Size (Mb)
PH	qPH-n-1	Gm03	4,390,000	12,070,000	7.68
	qPH-n-2	Gm03	15,860,000	30,880,000	15.02
	qPH-c1-3	Gm04	49,640,000	52,380,000	2.74
	qPH-k-2	Gm09	39,480,000	41,020,000	1.54
	qPH-k-3	Gm09	44,280,000	48,060,000	3.78
	qPH-o-5	Gm10	46,350,000	47,340,000	0.99
NNMS	qMS-c1-1	Gm04	50,350,000	52,380,000	2.03
	qMS-c2-3	Gm06	47,290,000	48,010,000	0.72
	qMS-c2-4	Gm06	48,040,000	48,040,000	0.00
	qMS-c2-5	Gm06	48,740,000	49,070,000	0.33
	qMS-c2-6	Gm06	49,350,000	50,520,000	1.17
	qMS-j-3	Gm16	2,010,000	5,360,000	3.35
	qMS-j-4	Gm16	31,390,000	37,880,000	6.49
	qMS-D2-1	Gm17	3,160,000	9,210,000	6.05

## Supplementary Materials

Supplementary materials can be found at https://www.mdpi.com/1422-0067/21/1/42/s1.

## Abbreviations

PH	plant height
NNMS	the number of nodes on main stem
CSSL	chromosome segment substitution line
QTL	quantitative trait loci
BSA	bulked segregant analysis
LOD	logarithm of odds
PVE	phenotypic variance explained of the QTL
ICIM	Inclusive Composite Interval Mapping
BR	Brassinolide
GA	Gibberellin
RT-qPCR	Real-time Quantitative PCR
SNP	Single Nucleotide Polymorphisms
MAS	marker-assisted selection
RIL	recombinant inbred lines
ED	Euclidean Distance
LD	linkage disequilibrium
KEGG	Kyoto Encyclopedia of Genes and Genomes
GO	gene ontology
