# Supplementary material for: Combined Linkage Mapping and BSA to Identify QTL and Candidate Genes for Plant Height and the Number of Nodes on the Main Stem in Soybean
Source: Int J Mol Sci. 2019 Dec 19;21(1):42. doi: 10.3390/ijms21010042 (PMC6981803; doi:10.3390/ijms21010042)
Supplement: Supplementary file 1 [file ijms-21-00042-s001.zip › Supplementary Tables.pdf]

Supplementary Tables

Table S1. The Correlation Analysis of PH and NNMS.

Years	Number	Pearson	Sig. (Bilateral)
2013	173	0.398**	0.000
2014	152	0.364**	0.000
2016	197	0.366**	0.000
BLUE	206	0.495**	0.000

Table S2. The statistic results of each sample SNP.

Traits	Total SNP	Multiple Alleles of SNP	Read < 4	Identical Genotypes of SNPs	High-Quality SNPs
**NNMS**	2664165	3905	0	444518	2215742
**PH**	3717836	8437	0	559012	3150387

Table S4. The information of candidate gene.

Gene ID	GM	Location (Mb)	Homologous Gene Symbol	Homologous Gene Description
Glyma.04G244200	GM04	51.17-51.21	AT5G51810	gibberellin 20 oxidase 2
Glyma.16G156700	GM16	31.53-31.71	AT3G54220	GRAS family transcription factor
Glyma.04G251900	GM04	50.35-52.38	AT4G08250	GRAS family transcription factor
Glyma.04G252300	GM04	50.35-52.38	AT1G77690	like AUX1 3
Glyma.04G254200	GM04	50.35-52.38	AT1G77850	auxin response factor 17

Table S5. Phenotypic data of PH and NNMS in 92 germplasm resource.

Variety	PH	NNMS
Heijiao-1-1161	74.2	12.6
Heihe-49	63.2	12.8
Hengdou-15	62.4	13
Bei-25031	72	13.2
Henong-95	66.8	14
Jiusan-14-70	69.6	14.4
Jinyuan-55	73.4	14.6
Heihe-52	76.6	14.6
NT-04	70.8	14.8
Boige dulotet geronne	89.8	14.8
Ha06-3869	90.8	14.8
Mushi-9	71.4	15
Heihe53-2016	76.2	15
Tuidou-8	79.4	15
Kennong-18	85	15
H09-95	71	15.2
Henong-73	74.2	15.2
Heihe-18	76.4	15.2
Dongsheng-7	82.4	15.2
Hefeng-51	67.6	15.4
Dongsheng-92	70.2	15.4
Heinong-35	88.4	15.8
Heihe-38	84.6	16
Heinong-44	88.4	16.2
Heihe-33	82	16.4
Heihe-50	82.4	16.4

Longqingdou-2	73.2	16.6
Longken-381	83.8	16.6
Wanglui	93.8	16.6
Ji94	98.2	16.6
777	78.8	16.8
Henong-85	82.2	16.8
Suinong-67	88.4	16.8
H10-2430	89.2	16.8
DN-L202	91.6	16.8
DND-253	95.6	16.8
Henong-62	99.6	16.8
Mu-05-026	70	17
Changnong-29	85.4	17
Beijiao-05-8021	88.4	17
Ken-09-1723	95.4	17
Fengshou-25	84.6	17.2
Heinong-56	90	17.2
q-15	91	17.2
K11-7456	115	17.2
Dongsheng-3	87.8	17.4
Beijiao-9206	92.8	17.4
Hefneg-25	95.2	17.4
L72-1241	80	17.6
Jiusanhei-05-59	103	17.6
NT-01	104.2	17.6
Shengdou-43	82.4	17.8
Dongda-2	98	17.8
Jike-1	108.6	17.8
Ke-C14-732	78.4	18
Ken-05-3762	111	18
Huajiang-4	92	18.2
Kenjian-28	98.4	18.2
M-39	102.4	18.2
Beidou-40	108	18.2
Shengdou-42	88.6	18.4
Beijiang-91	96.8	18.4
DN-76	80.2	18.6
Baojiao06-6046	108.6	18.6
Suinong07-1077	78.2	18.8
Suinong-68	91.2	18.8
Shujin05-9238	94	18.8
Fengdou-1	95.2	18.8
Heikang06-2	97.6	18.8
Ha-05-7778	98.2	18.8
L67-971	96	19
DN-88	92.8	19.2
Bindou-10	97.2	19.2
No.16	122.8	19.2
Kedou-28	108.4	19.4
L65-540	114.6	19.4
Chidou-1	97	19.6
LG2016-7	112.4	19.6
L73-79	134.2	19.6

Hefneg-40	94.8	19.8
Shuang-302	98.6	19.8
Suinong-69	108.6	20
L67-166	132.2	20
L72D-4045	140.4	20.8
H-39	116.8	21.4
Suinong-22	117	21.6
Hefeng-55	112.6	22
J-LG2016-1	112.8	22
Yapoche	123	22
Chi-16-04	119.2	23.6
Fendou-78	114.8	24
Magnolid	123.8	24.8

Table S6. Phenotypic statistics of PH and NNMS in 92 germplasm resource.

Phenotype	Min	Mix	Mean	SD	CV%	Skewness	Kurtosis
PH	62.40	140.40	93.07	16.90	18.16	0.52	−0.81
NNMS	12.60	24.80	17.59	2.41	13.70	0.50	0.65

Table S7. Candidate gene haplotype PH and MS in resource population.

Code	Candidate Genes	Haplotype Number	Main Haplotype	Main Haplotype Number	Main Haplotype Ratio
1	Glyma.04G244200	9	Hap_1	36	39.13%
			Hap_2	36	39.13%
			Hap_3	8	8.70%
2	Glyma.04G251900	23	Hap_2	9	9.78%
			Hap_5	55	59.78%
3	Glyma.10G223800	6	Hap_1	10	10.87%
			Hap_2	70	76.09%
4	Glyma.10G224900	4	Hap_1	60	65.22%
			Hap_2	24	26.09%
5	Glyma.16G156700	53	Hap_8	6	6.52%
			Hap_13	5	5.26%
			Hap_19	11	11.96%
			Hap_28	9	9.78%

Table S8. Phenotypic differences between excellent haplotypes.

Gene	Trait	Haplotype	Number	Mean ± SD	Haplotype	Number	Mean ± SD	*P* Value
Glyma.04G251900	PH	Hap_2	9	80.27 ± 13.54	Hap_5	55	97.07 ± 16.06	0.004
	MS	Hap_2	9	15.71 ± 2.23	Hap_5	55	17.84 ± 2.06	0.006
Glyma.16G156700	PH	Hap_13	5	109.48 ± 19.23	Hap_19	11	89.33 ± 8.26	0.005
		Hap_19	11	89.33 ± 8.26	Hap_28	9	102.98 ± 11.29	0.019

Table S9. The phenotypic statistics of extreme individual in PH and NNMS.

Extreme Performances	Variety	2016	2015	2014	2013
High PH	R148	118.75	107.20	99.80	120.56
	R94	103.40	116.20		
	R187	97.33	116.20		
	R200	98.60	101.50	108.80	110.63
	R156	95.67	94.20	102.00	120.00
	R61	110.00	83.25	103.00	111.67
	R55	97.40	106.40		
	R4	85.20		101.00	116.25
	R108	65.50		101.00	135.83
	R89	106.80	94.00		
	R176	91.80	91.50	101.00	116.25
	R169	103.80	83.60	101.75	110.00
	R157	91.40		95.67	110.44
	R165	96.00	98.00	88.50	113.57
	R21	102.20	91.40		102.22
	R114	95.00		96.60	102.14
	R166	86.80	92.60	98.00	112.50
	R158		94.33	87.60	110.44
	R203	81.80		89.00	120.00
	R124	100.25	92.67		
	R64	86.50	93.60	92.00	113.57
	R87	95.50	94.00		
	R26	91.25	97.80	84.80	102.22
	R86	80.00	86.20	98.20	111.11
	R19	91.00		85.80	103.33
	R206	89.20	94.20	89.20	100.63
	R109	91.00	92.20	83.50	106.00
	R17	84.00	94.20	89.20	100.63
	R215	93.60	77.80	91.00	105.50
	R22	103.25	86.80	80.00	97.56

Extreme Performances	Variety	2016	2015	2014	2013
Low PH	R103	64.80	71.20	76.40	85.75
	R130	77.25	55.00	86.60	79.29
	R146	62.80		81.80	78.44
	R40	69.20	75.25	64.60	88.00
	R105	73.00	75.50		
	R213	82.80	54.00	65.50	94.57
	R93	68.40	74.00	69.00	85.00
	R63	78.00	70.00		
	R91	66.25	75.20	69.00	85.00
	R192	73.00	74.67		
	R60	72.20		64.50	83.33
	R112	51.40		79.25	88.33
	R81	66.80	57.00	64.00	104.00
	R125	75.60	70.25		
	R134	64.40	77.00	65.75	83.33
	R58	64.60	82.67	60.00	82.50
	R52	57.60	77.60	62.00	91.88
	R88	65.80	60.60	70.80	88.13
	R75	78.50	61.60		

	R99	61.40	67.00	69.00	81.88
	R5	61.00		62.40	85.71
	R90	60.67	60.00	75.80	81.88
	R98	72.60	71.33	69.67	60.00
	R141	64.50	56.00	80.75	60.00
	R139	76.60	46.25	55.00	79.30
	R66	60.80	61.00	66.33	68.75
	R127	66.40	61.50	67.00	58.50
	R78	50.75		47.50	85.00
	R210	59.75	51.50	52.00	68.86
	R50	52.80	51.50	52.00	68.86

**Extreme Performances**	**Variety**	**2016**	**2014**	**2013**
High NNMS	R55	20		
	R158	14	25	20
	R76		22	16
	R148	17	22	18
	R156	16	17	24
	R109	18	16	21
	R15	17	19	
	R61	16	24	13
	R56	16	23	15
	R21	20		15
	R215	13	20	19
	R4	13	22	17
	R157	17	15	20
	R28	17	18	16
	R181	15	17	20
	R26	17	19	16
	R212	12	22	17
	R67	13	21	17
	R174	14	19	17
	R82	14	17	20
	R105	17		
	R191	13	16	21
	R213	16	12	22
	R172	14	17	18
	R17	16	18	15
	R169	16	19	14
	R43	16	17	16
	R175	14	18	17
	R10	18	17	14
	R165	16	16	16

**Extreme Performances**	**Variety**	**2016**	**2014**	**2013**
Low NNMS	R146	13	13	13
	R104	14	12	13
	R100	12	14	13
	R189	12	14	13
	R130	12	14	13
	R78	11	15	12
	R48	13		
	R167	13		
	R188	13	13	12

R185	12	12	14
R52	13	14	11
R60	12	15	11
R63	13		
R179	12		13
R182	12	13	13
R160	13	15	9
R91	13	13	11
R210	13	14	10
R33	12		13
R153	12	13	12
R143	11		13
R97	11	13	12
R96	11	14	11
R58	14	13	9
R88		12	11
R87	12		
R53	13		10
R50	11	14	10
R84	12		
R49	12		11

Table S10. Multiple Comparisons for PH of 5 materials for qRT-PCR.

(I) PH	(J) PH	Mean Difference (I-J)	Std. Error	Significant (I-J)	95% Confidence Interval	
					Std. Error	Mean Difference (I-J)
**R-19**	R-200	.20000	7.31720	.978	-14.4316	14.8316
	R-120	−61.85000*	7.76106	.000	−77.3692	−46.3308
	R-155	−66.00000*	7.31720	.000	−80.6316	−51.3684
	SN14	−27.21702*	5.44230	.000	−38.0996	−16.3345
R-200	R-19	−.20000	7.31720	.978	−14.8316	14.4316
	R-120	−62.05000*	7.76106	.000	−77.5692	−46.5308
	R-155	−66.20000*	7.31720	.000	−80.8316	−51.5684
	SN14	−27.41702*	5.44230	.000	−38.2996	−16.5345
R-120	R-19	61.85000*	7.76106	.000	46.3308	77.3692
	R-200	62.05000*	7.76106	.000	46.5308	77.5692
	R-155	−4.15000	7.76106	.595	−19.6692	11.3692
	SN14	34.63298*	6.02589	.000	22.5835	46.6825
R-155	R-19	66.00000*	7.31720	.000	51.3684	80.6316
	R-200	66.20000*	7.31720	.000	51.5684	80.8316
	R-120	4.15000	7.76106	.595	−11.3692	19.6692
	SN14	38.78298*	5.44230	.000	27.9004	49.6655
SN14	R-19	27.21702*	5.44230	.000	16.3345	38.0996
	R-200	27.41702*	5.44230	.000	16.5345	38.2996
	R-120	−34.63298*	6.02589	.000	−46.6825	−22.5835
	R-155	−38.78298*	5.44230	.000	−49.6655	−27.9004

* The Mean Difference is significant at the 0.05 level.

Table S11. Multiple Comparisons for NNMS of 5 materials for qRT-PCR.

(I) NNMS	(J) NNMS				95% Confidence Interval	
		Mean Difference (I-J)	Std. Error	Significant (I-J)	Std. Error	Mean Difference (I-J)
R-19	R-200	.80000	1.35312	.557	−1.9057	3.5057
	R-120	−3.45000*	1.43520	.019	−6.3199	−.5801
	R-155	−6.80000*	1.35312	.000	−9.5057	−4.0943
	SN14	−2.30638*	1.00641	.025	−4.3188	−.2939
R-200	R-19	−.80000	1.35312	.557	−3.5057	1.9057
	R-120	−4.25000*	1.43520	.004	−7.1199	−1.3801
	R-155	−7.60000*	1.35312	.000	−10.3057	−4.8943
	SN14	−3.10638*	1.00641	.003	−5.1188	−1.0939
R-120	R-19	3.45000*	1.43520	.019	.5801	6.3199
	R-200	4.25000*	1.43520	.004	1.3801	7.1199
	R-155	−3.35000*	1.43520	.023	−6.2199	−.4801
	SN14	1.14362	1.11433	.309	−1.0846	3.3719
R-155	R-19	6.80000*	1.35312	.000	4.0943	9.5057
	R-200	7.60000*	1.35312	.000	4.8943	10.3057
	R-120	3.35000*	1.43520	.023	.4801	6.2199
	SN14	4.49362*	1.00641	.000	2.4812	6.5061
SN14	R-19	2.30638*	1.00641	.025	.2939	4.3188
	R-200	3.10638*	1.00641	.003	1.0939	5.1188
	R-120	−1.14362	1.11433	.309	−3.3719	1.0846
	R-155	−4.49362*	1.00641	.000	−6.5061	−2.4812

"*" represents a significant difference at the 0.05 level; "**" represents an extremely significant difference at the 0.01 level.
