# Supplementary figures and images for: Combined Linkage Mapping and BSA to Identify QTL and Candidate Genes for Plant Height and the Number of Nodes on the Main Stem in Soybean
Source: Int J Mol Sci. 2019 Dec 19;21(1):42. doi: 10.3390/ijms21010042 (PMC6981803; doi:10.3390/ijms21010042)

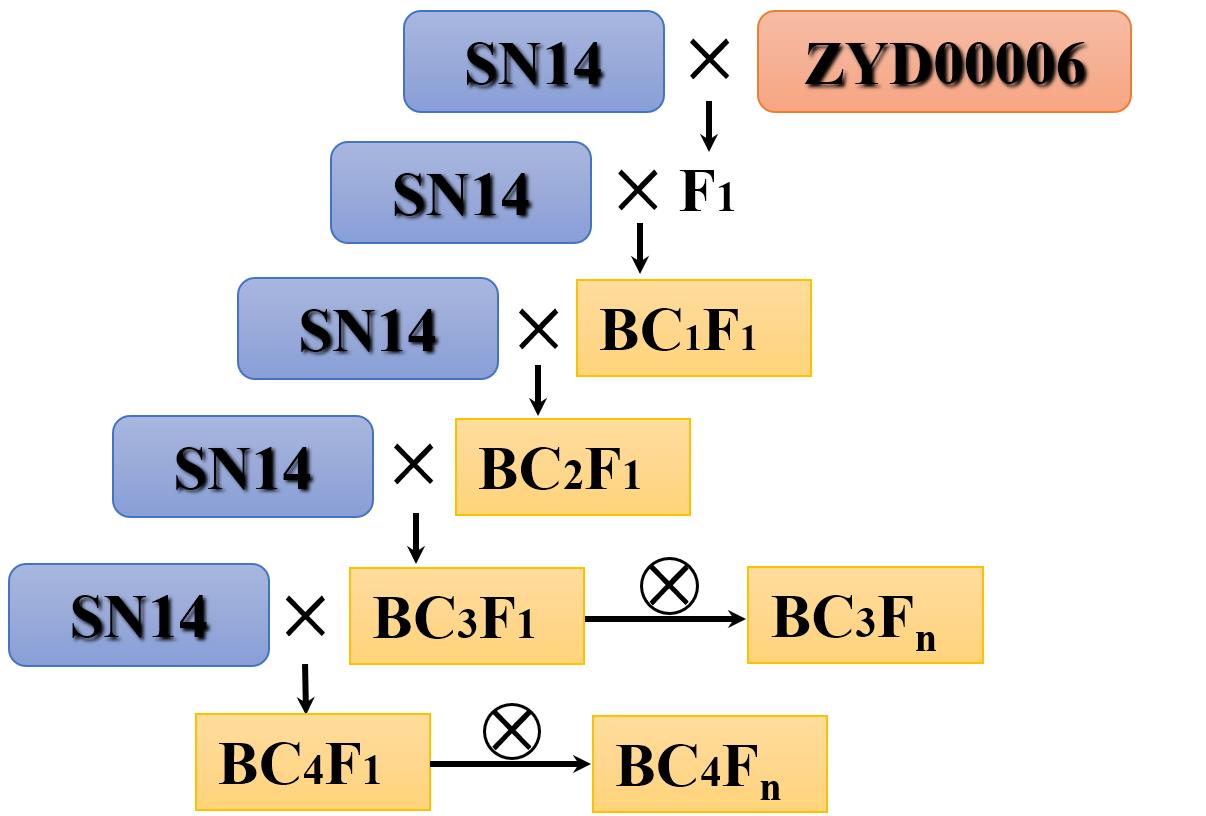

Supplement: Supplementary file 1 [file ijms-21-00042-s001.zip › Supplementary Figure S1.jpg]
